# Sodium and potassium intake in rheumatoid arthritis: a systematic review of clinical studies with implications for disease-related outcomes

**DOI:** 10.1007/s00296-026-06104-5

**Published:** 2026-04-01

**Authors:** Panagiota Anyfanti, Christina Antza, Konstantinos Tragiannidis, Andrej Belančić, Yusuf Ziya Şener, Andrea Katrin Faour, Alexandra Ainatzoglou, Elena Angeloudi, Evangelia Chaida, Theodoros Dimitroulas, Vasilios Kotsis

**Affiliations:** 1 3rd Department of Internal Medicine, Papageorgiou Hospital, Aristotle University of Thessaloniki, Thessaloniki, Greece; 2 Department of Basic and Clinical Pharmacology with Toxicology, Faculty of Medicine, University of Rijeka, Rijeka, Croatia; 3 Cardiology Department, Erasmus MC Thoraxcentrum, Rotterdam, ZH-Holanda Netherlands; 4 Independent researcher, Vancouver, BC Canada; 5 Fourth Department of Internal Medicine, Hippokration Hospital, Thessaloniki, Greece

**Keywords:** Arthritis, Rheumatoid, Sodium, Potassium, Cardiovascular diseases, Inflammation

## Abstract

Rheumatoid arthritis (RA) is characterized by immune-mediated inflammation resulting in excess cardiovascular disease (CVD) risk. Increased sodium, decreased potassium intake and their ratio have been acknowledged as CVD risk modifiers, while pathophysiological studies suggest a role in autoimmunity and inflammation. We aimed to review in a systematic manner the literature regarding the effects of dietary sodium, potassium intake and their ratio, on cardiovascular and disease-related outcomes in patients with RA. We performed a systematic literature search in Medline and Cochrane. Our research concluded to 1,283 patients (8 studies). Although not all studies coincide, most agree that increased dietary sodium and reduced dietary potassium intake are adversely associated with disease related (rheumatic and immunologic) outcomes. The ratio between sodium and potassium emerges a potential indicator of autoimmunity state that needs to be further balanced. Only 4 studies reported associations with cardiovascular outcomes in RA, particularly hypertension, although not consistently and none with hard CVD endpoints. These findings support that interventions promoting a poor in sodium and rich in potassium diet in RA may have beneficial effects in terms of autoimmunity regulation, suppression of inflammation and improvement in cardiovascular health.

**Supplementary Information:**

The online version contains supplementary material available at 10.1007/s00296-026-06104-5.

## Introduction

Rheumatoid arthritis (RA) is the most prevalent autoimmune inflammatory disease, affecting around 0.5-1% of the general population [1]. In 2020, an estimated 17.6 million people were living with RA globally, and this number is expected to increase by as much as 80.2% by 2050, thereby resulting in a considerably greater burden on health systems [2]. Atherosclerotic cardiovascular disease (CVD) represents the leading cause of death in patients with RA, who present an exaggerated CVD risk by as much as 50% compared to the general population [3]. Despite huge advancements in the CVD field, cardiac involvement remains frequently undiagnosed among patients with RA, largely because they are less likely to exhibit typical signs and symptoms compared to the general population [4]. Unfortunately, there are to date no widely available CVD risk calculators, either generic or RA-adapted, that can provide an accurate prediction of CVD risk in patients with RA [5]. Hence, simple markers that can be easily and affordably applied in routine clinical practice may be used as indirect indicators of CVD risk in RA.

Dietary habits is an important factor associated with high CVD risk. In the general population, a notable number of deaths from cardiovascular causes may be attributed to sodium consumption above a reference level of just 2.0 g per day [6]. Thereby, restriction in dietary sodium intake is a common recommendation in patients with high CVD risk and CVD comorbidities, such as hypertension and heart failure [7, 8]. However, scientific interest has gradually shifted to the beneficial effects of potassium intake, which is positively associated with beneficial CVD outcomes [9]. More recently, rather than either sodium or potassium consumption in separate, the ratio between sodium and potassium has been acknowledged as a potent mediator of CVD. Prospective cohort analysis has verified a dose-response association of higher sodium and lower potassium intakes, as estimated from urine excretion, with future cardiovascular risk [10, 11].

Based on the above, it could be extrapolated that dietary sodium and potassium intake may play an important role as a CVD predictor or mediator in patients with RA. In this group of patients, assessment of sodium and potassium intake could be additionally useful for their potential involvement in the course of the disease. There is preclinical data suggesting that sodium and potassium intake can influence the immune function and inflammation. High sodium consumption has been linked to increased pro-inflammatory responses and promotes T-cell activity, that can trigger autoimmune conditions like RA [12]. On the other hand, potassium intake has anti-inflammatory effects, modulating immune responses, that are induced by high sodium [13]. Although such data imply that the maintenance of a balance between sodium and potassium intake is essential, in order to regulate the inflammation and achieve immune balance, the extent to which these mechanisms are confirmed by clinical trials remains unclear.

Nevertheless, the role of sodium and potassium intake in patients with RA is still under investigation and there is no clear recommendation about their usage. Therefore, the objective of this study was to review in a systematic manner the literature regarding the effects of dietary sodium and potassium intake primarily on cardiovascular outcomes, and secondarily on disease-related outcomes in patients with RA.

## Methods

This systematic review was performed according to the Preferred Reporting Items for Systematic Reviews and Meta-Analyses (PRISMA) statement [14]. All research was conducted according to a protocol registered in the OSF database (available in https://osf.io/5watz/overview).

### Literature search strategy

We undertook searches through Embase Classic/Embase, Medline/Pubmed, and Cochrane Library, selective toward high-quality publications and containing most up-to-date information as per relevant recommendations for comprehensive searches, from inception to August 4, 2025 [15]. We used search terms that had been identified from initial scoping searches and target references, to identify studies evaluating the association of sodium and/or potassium intake with cardiovascular and/or disease-related outcomes. Search terms included, but not limited to, RA, sodium or salt intake (dietary recall, interviews or diet records; urinary sodium excretion in spot or 24 h urine samples), potassium intake (dietary recall, interviews or diet records; urinary potassium excretion in spot or 24 h urine samples), urinary sodium-to-potassium ratio (spot or 24 h urine samples), cardiovascular outcomes (cardiovascular events; arterial stiffness; pulse wave velocity; carotid atherosclerosis; intima-media thickness), disease-related outcomes (disease duration, disease activity, disease activity score in 28 joints: DAS28, inflammatory markers, C-reactive protein: CRP, erythrocyte sedimentation rate: ESR, proinflammatory cytokines). Conference abstracts and references of relevant studies and systematic reviews were perused and experts were contacted in order to identify any possible available study.

### Eligibility and exclusion criteria

We included original, cross-sectional or case-control or cohort studies examining the impact of sodium and potassium intake on cardiovascular and disease-related outcomes. Inclusion criteria were: (a) established RA, (b) estimation of dietary sodium/salt and potassium intake (as assessed from dietary recall or weighed diet records; urinary sodium excretion, urinary potassium excretion and urinary sodium-to-potassium ratio measured from spot or 24 h urine samples), (c) estimation of cardiovascular outcomes (major adverse cardiovascular events; surrogate markers of cardiovascular disease: pulse wave velocity, carotid intima-media thickness), (d) estimation of disease-related outcomes (disease duration and activity, inflammatory markers, proinflammatory cytokines). Exclusion criteria were: (a) studies with less than 10 individuals, (b) studies not reporting on vascular or disease-related outcomes, (c) basic research (animal or cellular studies), (d) studies not published in English.

### Screening process

Two blinded investigators (A.B., Y. Z. S.) screened the results of the algorithm for eligibility at the level of title and abstract, after duplicate removal. In case of disagreement, consensus was reached with a third senior reviewer (A. K. F.). The remaining articles were assessed at full-text level, and in case of disagreement, a third senior reviewer assisted (C. A.).

### Data extraction and quality assessment in individual studies

Data were extracted from the studies included in the current systematic review by the two investigators in an unblinded manner. The data extracted included name of first author and year of the study, study design and intervention, total number of patients enrolled in each study, % or number of males/females, mean age, inflammatory markers (ESR, CRP), DAS28, positivity for rheumatoid factor, endpoints assessed, cardiovascular, rheumatic and immunological outcomes. The Cochrane Risk of Bias 2 tool was used to assess the risk of bias in the randomized controlled trial, while the Newcastle-Ottawa scale was used to assess the quality of the cohort and cross-sectional observational studies [16, 17].

## Results

**Fig. 1:**
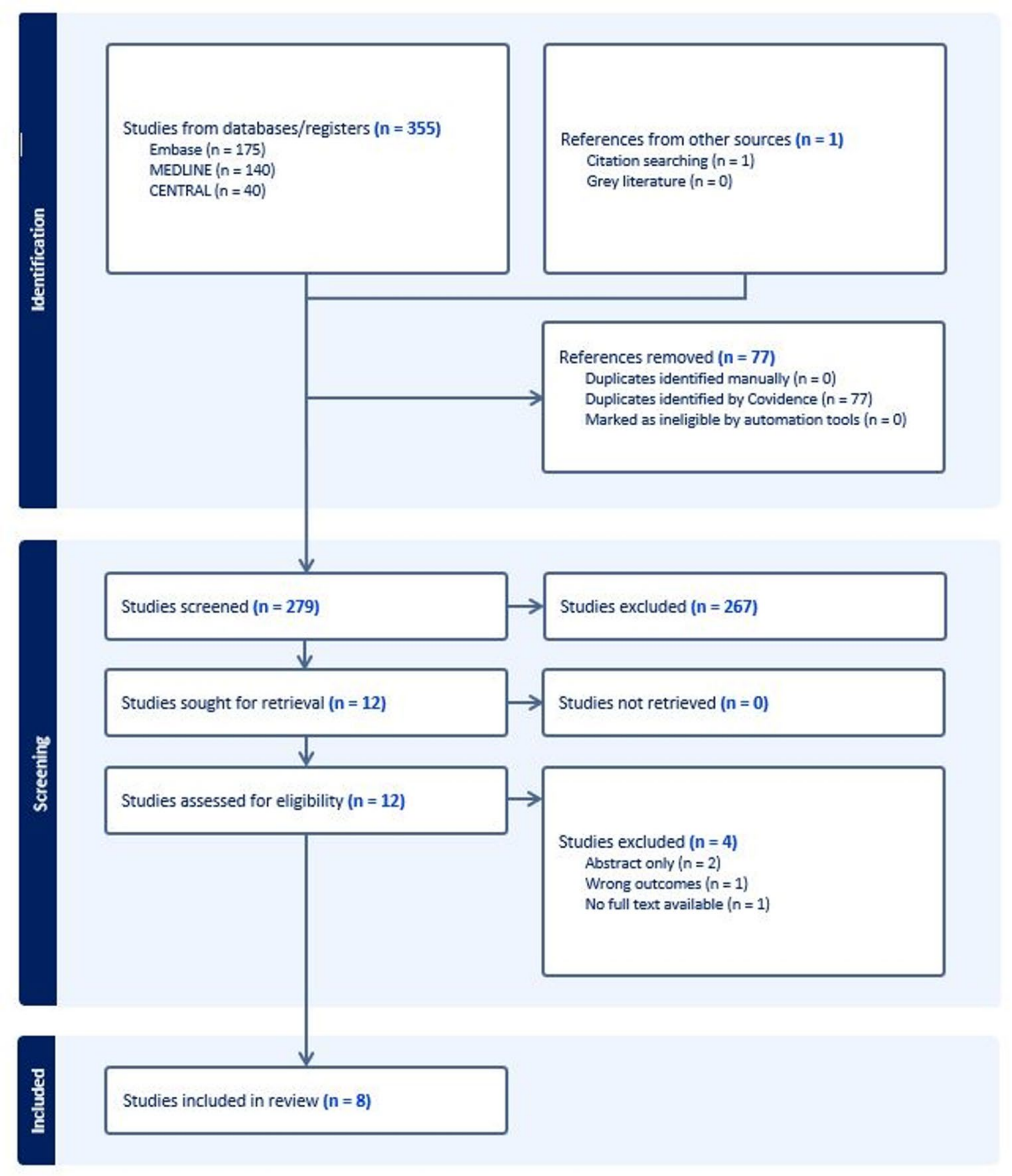
PRISMA flowchart of the included studies

Our search concluded to 1,283 patients. Only 8 studies met the inclusion criteria and were included in the systematic review. Figure 1 summarizes the selection procedure in a PRISMA flowchart.

The characteristics of the studies included in our systematic review are shown in Table 1. Studies were conducted in Europe (3 studies), America (2 studies) or Asia (3 studies), and were published between 2014 and 2025. The design of the studies was prospective, observational in 1 study, case-control in 3 studies, observational/cross-sectional in 3 studies, and only one study was randomized, single blind, single center study. The number of patients included in the studies ranged from 29 to 336.

**Table 1:** Patients’ baseline characteristics

Study	Country	Study population	All cases			RA cases					
			Total patients, *n*	Sex, (F/M)	Age, years	RA cases, *n*	Age, years	Sex, (F/M)	CRP, mg/L	DAS28	RF (+), *n* (%)
Scrivo et al. [18]	Italy	RA cases + SLE cases	29	28/1	N/A	14	58.5	13/1	0.5	3.2	10 (71.4)
Marouen et al. [21]	France	RA cases + healthy controls	48	34/14	58	24	58	17/7	17	4.1	14 (58.3)
Carranza-Leon et al. [22]	USA	RA cases + controls	258	172/86	53.5	166	54	114/52	N/A	3.9	N/A
Vitales-Noyola et al. [20]	Mexico	RA cases + SLE cases	75	70/5	39.5	38	42.5	36/2	N/A	N/A	N/A
Minamino et al. [13]	Japan	RA cases	336	279/57	61.8	336	61.8	279/57	1	2.4	N/A
Kianifard et al. [19]	India	RA cases	172	153/19	48.2	172	48.2	153/19	280.3	N/A	130 (75.6)
Kianifard et al. [23]	India	RA cases + healthy controls	304	N/A	40.7	139	46.3	110/29	N/A	N/A	N/A
Anyfanti et al. [24]	Greece	RA cases	61	48/13	62.4	61	62.4	48/13	3.2	3.2	N/A

The quality of assessment of the included studies is presented in Supplementary Table. In general, the methodological quality of the included observational studies was moderate to high. According to the Newcastle–Ottawa Scale, quality scores for cohort studies ranged from 6 to 8, and for case-control studies between 7 and 8 points, out of a maximum of 9 points. Most studies demonstrated good representativeness of study populations and adequate ascertainment of exposure and outcomes. Reduced scores were mainly related to limitations in the comparability arm and, for cohort studies, to incomplete follow-up. The single randomized controlled trial was judged to raise some concerns overall according to the Cochrane Risk of Bias 2 tool, primarily related to the randomization process and potential deviations from intended interventions, although outcome measurement was considered at low risk of bias.

### Disease-related outcomes

All 8 studies assessed rheumatic and/or immunologic outcomes. Only 2 studies were interventional. In the study of *Scrivo et al.*, RA patients were prospectively studied, having undergone a 3-week restriction of dietary sodium intake, reflected by a drop in their 24-hour sodium urine excretion under 85 mEq/die and subsequently returning to a 2-week period of normal sodium intake [18]. Upon sodium restriction, levels of Th17 cells followed a downward trend, while Treg cells tended to increase, with both effects being reversed during normal sodium intake and none of them reaching significance. Apoptotic and cellular proliferation markers remained unaffected by dietary restrictions, while serum TGFβ1 and IL-9 were significantly suppressed at the end of the study compared to baseline [18]. *Kianifard et al.* further studied the effects of potassium supplementation on chronic symptomatic RA patients [19]. Maximum disease improvement and joint pain alleviation was reported in the subgroup maintaining a vegetarian diet while receiving a well-tolerated potassium food supplement. This outcome was retrieved by comparing the pain visual analogue scale scores of patients at week 16 post-intervention compared to baseline, which demonstrated a significant mean change, significantly superior to a plain vegetarian diet or routine diet [19].

The rest of studies had a cross-sectional design. In the study of *Vitales et al.*, RA patients were classified as low sodium intake or high sodium intake upon their sodium intake being less or exceeding 5 g/day, respectively [20]. In both groups, the populations of CD4 + CD25^high^Foxp3+ and CD69 + Treg cells were significantly limited, while Th17 cells were increased compared to healthy subjects, irrespective of sodium consumption patterns. Nonetheless, sodium intake was inversely correlated with the CD69 + Treg cell subset of RA patients. Disease activity measured with DAS28 did not differ between the two groups, while Treg-mediated cytokine secretion was equally suppressed and Th17 differentiation remained unaffected by sodium intake [20].

In the study of *Marouen et al.*., sodium excretion of early RA patients was reported to be significantly elevated compared to controls, even when adjusted for confounding factors such as tobacco, anti-hypertensive regimen and NSAIDs, as well as alimentary factors using the food frequency questionnaire (FFQ) [21]. Daily sodium excretion levels were higher in RA patients with erosions at diagnosis than without, while the presence of auto-antibodies and RF positivity did not have such impact. Lastly, disease activity as measured by DAS28-CRP was not correlated with sodium excretion patterns [21].

Conflicting evidence was documented in the study of *Carranza-Leon et al.*, in which sodium excretion levels estimated with the Kawasaki formula were found equally elevated in both RA patients and controls [22]. Opposingly, the Na/K excretion ration was significantly greater in RA than in controls. Neither sodium nor potassium intake patterns were correlated with inflammatory markers or the DAS28 index [22], in line with the aforementioned studies [20, 21]. These results are in line with the preliminary observations by *Kianifard et al.*, who found non-significant correlations between dietary potassium with pain visual analogue scale (VAS) scores, Health Assessment Questionnaire (HAQ) score and serum cortisol. No association was reported between dietary potassium and several other clinical variables (including joint counts pain/tenderness and swelling, ESR, CRP and DAS28). However, dietary potassium intake in RA patients was significantly low especially in women, who consumed less potassium compared to men [23].

By contrast, in the study of *Minamino et al.*, spot urine Na/K ratio was found significantly indicative of disease activity, regardless of gender or prednisolone intake [13]. Furthermore, in the study by Anyfanti et al., disease activity as reflected by ESR and DAS28 levels was inversely related to urinary K excretion, while no such association was found with urinary Na or urinary Na/K ratio [24]. This evidence is in favor of the potential capacity of dietary sodium restriction to curb disease progression in RA.

### Cardiovascular outcomes

Only 4 studies assessed the association of sodium and potassium intake with cardiovascular outcomes, mostly focusing on hypertension. *Carranza-Leon et al.* reported higher prevalence of hypertension, pointing towards increased salt sensitivity, in the RA group compared to controls [22]. Notably, levels of potassium excretion were inversely associated with diastolic blood pressure of RA patients. Although the Na/K excretion ration was significantly greater in RA than in controls, it was not associated with hypertension or metabolic syndrome comorbidities [22]. By contrast, urinary Na/K ratio displayed a positive correlation with both systolic and diastolic blood pressure of patients, as well as independent association with hypertension onset, in the study of *Minamino et al.* [13]. In the prospective interventional study by *Kianifard et al.*, a downward trend was recorded in mean blood pressure levels of patients under potassium-rich diet, without however reaching significance [19].

Lastly, Anyfanti et al. assessed myocardial perfusion with subendocardial viability ratio, and arterial stiffness with pulse wave velocity (PWV) and augmentation index (AIx), as surrogate cardiovascular indices in patients with RA [24]. Levels of 24 h urinary sodium excretion were significantly correlated with HDL-c and uric acid levels, eGFR was proportional to urinary potassium excretion, while urinary Na/K ratio was significantly associated with all three lab markers. Although PWV and AIx were not correlated with urinary sodium or potassium levels, SEVR was inversely associated with both urinary sodium and urinary Na/K ratio, even when adjusted for cardiovascular risk factors [24].

## Discussion

In the present systematic review, we evaluated the effects of the dietary sodium and potassium intake on cardiovascular and disease-related outcomes in patients with RA. We were able to identify only 8 studies assessing the association with rheumatic or immunologic parameters, of which only 4 studies provided further information for the association with cardiovascular outcomes. The heterogeneity in outcomes did not allow for further quantitative synthesis of the results. However, qualitative analysis of the results in this systematic review generates important considerations that need to be addressed in future research.

Remarkably, available evidence suggests that sodium and potassium intake may modulate cardiovascular outcomes in patients with RA, although results are limited and inconclusive. This is mostly the case for hypertension, with studies indicating that the dietary potassium intake and the balance between sodium and potassium have a key-role in the regulation of blood pressure in RA [13, 19, 22]. No study has used hard cardiovascular endpoints. However, sodium intake and Na/K ratio were associated with myocardial perfusion in one study [24], further supporting the notion that dietary modification of both sodium and potassium may have favorable effects on the cardiovascular system in patients with RA. This hypothesis is supported from well-established data in the general population and in high-risk patients. More specifically, high sodium intake has been associated with increased plasma volume and cardiac output, hypertension, heart failure, and major adverse CVD outcomes, which are decreased by interventions aiming at salt restriction [25–27]. On the other hand, high potassium intake has shown beneficial effects on the cardiovascular system, such as blood pressure reduction, improvement in vascular function, and decrease in CVD events [10, 11, 28, 29]. Hence, urinary Na/K ratio may emerge as potential CVD risk indicator in patients with RA, whose CVD risk estimation remains problematic, but this hypothesis needs to be tested in future studies in the absence of relevant data. Considering that patients with RA may present subclinical vascular damage even in the absence of overt CVD manifestations [30–32], appropriately designed studies are needed to determine further whether optimizing Na/K intake may be beneficial in terms of CVD health.

Beyond cardiovascular outcomes, several disease-related rheumatic and immunological effects may be affected as suggested by relevant studies in the present systematic review. Although more data exist, their evaluation is similarly complicated, given the type and divergent methodology of the existing studies. However, there is some evidence summarized in Table 2 that excessive sodium intake is not helpful in the autoimmunity and is associated with more severe disease characteristics affecting RA physiology [18, 21, 22]. Likewise, potassium intake appears to be associated with lower inflammation and disease activity [19, 23, 24], while Na/K ratio is also clinically relevant in RA, as it has an impact on presentation of the disease [13, 20]. Last, both studies by *Kianifard et al.*, present findings that encourage the use of dietary potassium as a modulator of disease activity [19, 23]. Despite objective difficulties to draw firm conclusions linked to divergent study methodology, there is an overall agreement that maintaining a balance between sodium and potassium intake may be important for effective disease control. Such strategies could potentially have a notable clinical and immunologic impact on patients with RA, but this hypothesis requires further verification in clinical research.

**Table 2:** Study characteristics including study type, study intervention, and clinical outcomes (cardiovascular, rheumatic and immunological outcomes)

Study	Study type	Intervention	Endpoint	Cardiovascular outcomes	Rheumatic & Immunologic outcomes
Scrivo et al. [18]	Prospective, observational	Restriction of dietary Na intake	Changes in the frequency and functions of Th17 and Treg cells	N/A	Th17 cells: A trend of decreasing during low-sodium diet and an increase after return to normal sodium Treg cells: A trend of increase during low-sodium diet and decrease following return to normal sodium A significant reduction in serum levels (pg/ml) of both TGFβ1 [3002 (2193–37069) vs. 49069 (38221–58005); *p* = 0.0016) and IL-9 [1.65 (0-17.5) vs. 12.8 (3-41.2); *p* = 0.0007)
Marouen et al. [21]	Case-control study	Sodium intake (24-hr urinary sodium excretion)	Comparing Na excretion in patients with RA and controls Assessing the association between Na excretion and disease activity	N/A	Increased sodium excretion for patients with early RA (2,849 ± 1,350 vs. 2,182 ± 751.7 mg/ day, *p* = 0.039) than controls. Increased sodium excretion for patients with radiographic erosion at the time of diagnosis than those without (*p* = 0.028) No correlation between Na excretion and disease activity (DAS28-CRP and CRP) Similar Na excretion in RF (+) and RF (-) cases
Carranza-Leon et al. [22]	Case-control study	Na and K excretion (24-hr urinary excretion)	Comparing Na and K excretion between RA cases and controls Assessment of association between Na and K excretion and blood pressure in RA	No significant association between Na + and K+ excretion and systolic blood pressure among RA patients. Inverse association between K+ excretion and diastolic blood pressure (adjusted β=−1.79, *p* = 0.04).	Higher Na: K excretion ratio in RA compared to controls [2.0 (1.6–2.4) vs. 1.7 (1.5–2.1)]. No significant association between Na + or K+ excretion and DAS28, CRP, IL-6, IL-10, VCAM-1.
Vitales-Noyola et al. [20]	Observational study	Low salt intake (LSI < 5.0 g/day) and high salt intake (HIS-≥5.0 g/day)	Impact of LSI- vs. HSI on levels of CD4 + CD25+ Foxp3 + and CD4 + CD69+ Foxp3 − Treg cells	N/A	Negative association between CD4 + CD69+ Foxp3 − Treg cells and sodium intake in RA cases Similar suppressor activity of CD4 + CD25+ Foxp3 + and CD4 + CD69+ Foxp3 − Treg cells in LSI and HSI patients or controls
Minamino et al. [13]	Cross-sectional study	Salt load index (Urinary Na: K ratio)	Impact of salt intake on hypertension and disease activity outcomes	Urinary Na: K excretion ratio is correlated with both systolic (*r* = 0.1516, *p* = 0.0054) and diastolic (*r* = 0.1173, *p* = 0.0316) blood pressure Higher urinary Na: K excretion ratio in RA patients with hypertension than cases without hypertension Urinary Na: K ratio is a predictor of hypertension in RA patients (OR 1.34, 95% CI 1.13– 1.57)	Urinary Na: K ratio is independently and positively associated with DAS28- ESR (estimate 0.12, *p* < 0.001) Urinary Na/K ratio is independently associated with DAS28-ESR in gender-separated analysis as well as in prednisolone separated analysis.
Kianifard et al. [19]	Randomized, single blind, single center study	Potassium rich vegetarian diet and potassium supplementation	Impact of potassium supplementation on joint pain in 3 arms (K rich diet + K supplement; K rich diet; Normal diet)	Changes in blood pressure at 16th week compared to baseline: K rich diet + K supplement group: 129/80 mmHg ◊116/80 mmHg K rich diet group: 131/79 mmHg◊ 117/80 mmHg Normal diet: 125/79 mmHg◊ 126/82	The mean change in pain visual analogue scale (− 2.23, 95% CI − 2.99 to − 1.48) at week 16 (primary efficacy) from baseline was superior in K rich diet plus K supplement group compared to both cases with K rich diet only (*p* = 0.004) and controls with normal diet (*p* = 0.002). Significant association between high daily potassium intake (5–7.5 g) with low pain (VAS ≤ 4 cm) (OR:3.14)
Kianifard et al. [23]	Case-control study	Measurement of dietary K intake	Comparing the dietary K intake in RA cases with controls and assessing the impact of dietary K intake on RA disease activity	N/A	Mean daily dietary K + was 1238.7 mg (± 770.3) in RA and 3334.9 mg (± 756.3) in controls (*p* < 0.001) Weak but non-significant correlation of dietary K with pain VAS scores, HAQ score and serum cortisol; no correlation with joint pain/tenderness and swelling, ESR, CRP and DAS 28
Anyfanti et al. [24]	Observational study	Na and K excretion (24-hr urinary excretion)	Assessment of the association between Na, K intake and Na: K ratio in urine with: Myocardial perfusion and vascular stiffness Inflammation and disease-related parameters	Myocardial perfusion inversely correlated with both urinary Na (*r* = − 0.290, *p* = 0.027) and urinary Na: K ratio (*r* = − 0.270, *p* = 0.041), yet not with urinary K (*r* = − 0.014, *p* = 0.914), confirmed in multivariate analysis. Neither PWV nor AIx was associated with urinary Na, urinary K excretion, or their ratio.	Neither urinary Na excretion nor urinary Na: K ratio was associated with ESR, CRP, disease duration, and DAS28. Urinary K excretion was inversely associated with ESR (*r* = − 0.269, *p* = 0.039) and DAS28 (*r* = − 0.387, *p* = 0.003).

Previous publications have focused on dietary components as a risk factor for developing RA. In the Västerbotten Intervention Program (VIP) cohort, sodium intake more than doubled the risk for RA among smokers, but this association was not observed among non-smokers [33]. Using data from the same cohort, no significant associations were observed between diet, assessed as food groups, as macronutrients and as scores of dietary patterns, or alcohol consumption, and the risk of development of RA after additional adjustment for sodium intake [34]. In a recently published study, no significant associations were observed between the risk of RA and dietary intakes of sodium or potassium among UK Biobank participants, although high intakes of potassium and sodium were significantly associated with RA among NHANES participants. Insufficient exposure levels in the population or potential underestimation of sodium intake in dietary recalls may act as significant confounders to these results [35].

Although clinical data offer only limited or inconclusive support for this mechanistic narrative, there are some findings from pathophysiological and pre-clinical studies in favour of the hypothesis that sodium and potassium intake may be important for effective disease control. Experimental studies highlight that pathologic differentiation of Th17 cells is enhanced by high sodium intake, which further suppresses the development and function of regulatory T cells (Tregs) is suppressed [36]. Given that Tregs are responsible for immune tolerance, this leads to the formation of a Th17 immune profile, which subsequently triggers the production of pro-inflammatory cytokines [37]. Hence, high sodium can be related to increased inflammatory signaling or more active disease state in patients with autoimmune conditions including RA. On the other hand, potassium appears to have an anti-inflammatory role, possibly with a milder influence to the autoimmunity per se, thereby accompanied by potential benefits in the reduction of pain and disease activity. Pathophysiologically, potassium controls the intracellular ionic gradients and the signaling pathways in immune cells. This leads to the T-cell activation and cytokine production associated with the stabilization of the immune responses and the limitation of the inflammatory load [38]. Thereby, potassium channels have been proposed as therapeutic targets for autoimmune disorders [39], Based on the above, an impaired Na/K ratio may shift the immune cell balance toward inflammation, with a high ratio related to higher inflammatory burden, and a lower ratio promoting a balanced immune environment.

### Strengths and limitations

This is the first effort to address in a systematic manner an emerging novel area of future research, e.g., the association of sodium and potassium intake with cardiovascular and disease-related outcomes. The solid methodological approach that was followed reinforces the scientific reliability of the review. Most studies were of moderate-to-high quality. Limitations of the present systematic review are inherent to the methodological design of the included studies, particularly the cross-sectional design of the majority of studies that does not allow for conclusions regarding causality. The review was restricted to studies published in peer-reviewed journals in the English language. Although this approach was chosen to ensure methodological quality, reproducibility, and accurate data extraction, it may have introduced language and publication bias. Only eight relevant articles were identified, which is a limited number for a systematic review. Studies were highly heterogeneous in terms of design, exposure measurement, and outcomes. In addition, several confounders may influence urinary sodium and potassium excretion such as renal function, the use of antihypertensives (especially diuretics), and RA–specific factors such as corticosteroid and NSAID use, and future studies are needed to systematically report and adjust for these confounders. Finally, the included studies assessed different outcomes applying divergent methodology, and no hard CVD endpoints were evaluated. For these reasons, statistical analysis was not feasible. Although this does not alter or degrade the results and their analysis, it highlights the need for the conduction of appropriately designed studies to explore these relationships more thoroughly and assess the potential clinical implications of the observed associations.

## Conclusion

In conclusion, a limited number of clinical studies exists regarding the association between sodium, potassium intake and their ratio with cardiovascular and disease-related outcomes, with notable heterogeneity in the applied methodology and assessed endpoints. Nevertheless, the assessment of the existing findings already provides some potentially important key-notes. Although not all studies conducted in RA coincide, most agree that increased dietary sodium intake and reduced dietary potassium intake can have disadvantages on rheumatic and immunologic outcomes, with probable negative effects on cardiovascular aspects, particularly hypertension. Pending confirmation from appropriately designed studies with hard CVD outcomes, this hypothesis could be of importance for CVD risk modification in patients with RA, who present increased CVD risk compared to the general population. Given that the balance between sodium and potassium intake is a simple ratio that can be easily measured in routine clinical practice, its potential role as an indicator for the risk of autoimmune disease progression warrants further investigation, and the effects of interventions aiming at optimizing Na/K ratio on cardiovascular health need to be explored.

## Supplementary Information

Below is the link to the electronic supplementary material.

Supplementary Material 1

## Data Availability

Data sharing is not applicable to this article; no new datasets were generated during the current study.
