# Supplementary material for: Sodium and potassium intake in rheumatoid arthritis: a systematic review of clinical studies with implications for disease-related outcomes
Source: Rheumatol Int. 2026 Apr 1;46(4):69. doi: 10.1007/s00296-026-06104-5 (PMC13038458; doi:10.1007/s00296-026-06104-5)
Supplement: Supplementary file 1 — Supplementary Material 1 [file 296_2026_6104_MOESM1_ESM.docx]

| **Supplementary Table. Quality assessment of the included studies** | | | | | | | | | | | | | | | |
| --- | --- | --- | --- | --- | --- | --- | --- | --- | --- | --- | --- | --- | --- | --- | --- |
| **(a) Quality assessment of the included non-randomized cohort studies via Newcastle-Ottawa scale (case-control studies)** | | | | | | | | | | | | | | | |
| **Publication** | **Selection** | | | | | | | **Comparability** | | **Outcome** | | | | **Quality score (maximum=9)** | |
|  | **Representativeness of the exposed cohort** | | **Selection of the non- exposed cohort** | | **Ascertainment of the exposure** | | **Outcome status at start of study** |  |  | **Assessment of the outcome** | **Length of follow-up** | | **Adequacy of follow-up** |  |  |
| Scrivo et al. (2017) | ***** | | **-** | | ***** | | ***** | ****** | | ***** | ***** | | ***** | **8** | |
| Minamino et al. (2021) | ***** | | **-** | | ***** | | **-** | ****** | | ***** | ***** | | **-** | **6** | |
| Anyfanti et al. (2025) | ***** | | **-** | | ***** | | ***** | ***** | | ***** | ***** | | **-** | **6** | |
| **(b) Quality assessment of the included non-randomized case-control studies via Newcastle-Ottawa scale (cohort studies)** | | | | | | | | | | | | | | | |
| **Publication** | **Selection** | | | | | | | **Comparability** | | **Outcome** | | | | **Quality score (maximum=9)** | |
|  | **Representativeness of cases** | | **Selection of controls** | | **Adequate case definition** | | **Definition of controls** |  |  | **Ascertainment of exposure** | **Same method for cases and control** | | **Non-response rate** |  |  |
| Kianifard et al. (2014) | ***** | | ***** | | ***** | | ***** | ****** | | ***** | ***** | | **-** | **8** | |
| Marouen et al. (2017) | ***** | | ***** | | ***** | | ***** | ****** | | ***** | ***** | | **-** | **8** | |
| Carranza-Leon et al. (2017) | ***** | | ***** | | ***** | | ***** | ***** | | ***** | ***** | | **-** | **7** | |
| Vitales-Noyola et al. (2018) | ***** | | ***** | | ***** | | ***** | ***** | | ***** | ***** | | **-** | **7** | |
|  |  | |  | |  | |  |  | |  |  | |  |  | |
| **(c) Quality assessment of the included randomized controlled trials via Cochrane risk of bias 2 tool** | | | | | | | | | | | | | | | |
| **First author**  **Year of publication** | | **Randomization process** | | **Deviations from the intended interventions** | | **Missing outcome data** | | | **Measurement of outcomes** | | | **Selection of the reported result** | | | **Overall** |
| Kianifard et al. (2024) | | Some concerns | | Some concerns | | Some concerns | | | Low risk | | | Some concerns | | | Some concerns |
